# Subclinical infection occurs frequently following low dose exposure to prions by blood transfusion

**DOI:** 10.1038/s41598-022-15105-w

**Published:** 2022-06-28

**Authors:** M. Khalid F. Salamat, Paula Stewart, Helen Brown, Kyle B. C. Tan, Allister Smith, Christopher de Wolf, A. Richard Alejo Blanco, Marc Turner, Jean C. Manson, Sandra McCutcheon, E. Fiona Houston

**Affiliations:** 1 Royal (Dick) School of Veterinary Studies, The Roslin Institute, University of Edinburgh, Easter Bush, Midlothian, Edinburgh, UK; 2 Scottish National Blood Transfusion Service (SNBTS), The Jack Copland Centre, Edinburgh, UK

**Keywords:** Microbiology, Diseases, Neurology, Pathogenesis

## Abstract

Infectious prion diseases have very long incubation periods, and the role that subclinical infections play in transmission, persistence and re-emergence of these diseases is unclear. In this study, we used a well-established model of vCJD (sheep experimentally infected with bovine spongiform encephalopathy, BSE) to determine the prevalence of subclinical infection following exposure by blood transfusion from infected donors. Many recipient sheep survived for years post-transfusion with no clinical signs and no disease-associated PrP (PrP^Sc^) found in post mortem tissue samples by conventional tests. Using a sensitive protein misfolding cyclic amplification assay (PMCA), we found that the majority of these sheep had detectable PrP^Sc^ in lymph node samples, at levels approximately 10^5^–10^6^ times lower than in equivalent samples from clinically positive sheep. Further testing revealed the presence of PrP^Sc^ in other tissues, including brain, but not in blood samples. The results demonstrate that subclinical infection is a frequent outcome of low dose prion infection by a clinically relevant route for humans (blood transfusion). The long term persistence of low levels of infection has important implications for prion disease control and the risks of re-emergent infections in both humans and animals.

## Introduction

Prion diseases are invariably fatal neurodegenerative disorders, including bovine spongiform encephalopathy (BSE) in cattle, scrapie in sheep, chronic wasting disease (CWD) in cervids, and Creutzfeldt-Jakob disease in humans. The hallmark of prion diseases is the conversion of a host encoded prion protein, PrP^C^ into a misfolded partially protease resistant form, termed PrP^Sc^. According to the prion hypothesis, PrP^Sc^ is also the infectious agent responsible for transmission of infection between susceptible hosts^1^. Due to the lack of a nucleic acid genome, it is postulated that heritable phenotypic differences between prion strains are enciphered by conformational variants of PrP^Sc^^2^. The distribution of PrP^Sc^ in tissues of affected individuals varies depending on the host species and prion agent or strain. In some diseases (e.g. sporadic CJD, BSE), PrP^Sc^ is mainly detected within the central and peripheral nervous systems, whereas in others (e.g. scrapie, CWD, variant CJD) PrP^Sc^ is also readily detectable in a range of secondary lymphoid tissues e.g. spleen, tonsil, lymph nodes^3–6^.

The zoonotic transmission of BSE led to the emergence of variant Creutzfeldt-Jakob disease (CJD), a novel human prion disease, during the 1990s in the UK^7^. Measures taken to prevent transmission in cattle and exposure of the human population to infected meat appear to have been successful in controlling vCJD, with a total of 178 cases identified to date in the UK, and the peak incidence occurring in 2000. However, it is estimated that millions of people were exposed to BSE contaminated meat products, and the true burden of infection in the human population remains unclear. Concerns about the possibility of hidden or subclinical vCJD infection were heightened by the demonstration that cross-species transmission of hamster-adapted scrapie to mice resulted in prolonged persistence and replication of prions in the absence of clinical signs^8,9^. In addition, it has been shown using transgenic mouse models that cross-species transmission of prions such as CWD and BSE can result in more frequent infection of spleen than brain (without clinical signs), suggesting that lymphoid tissues are more permissive than brain to replication of prions from other species^10^.

In order to estimate the extent of vCJD infection in the UK population, several large-scale anonymized surveys of human tonsil and appendix samples removed during surgery have been conducted to look for evidence of abnormal PrP^Sc^ deposition in these lymphoid tissues. One of the largest surveys of birth cohorts likely to have had dietary exposure to BSE found PrP^Sc^ deposition in sixteen out of 32,441 appendix samples, leading to an estimated prevalence of abnormal PrP in up to 1 in 2000 of the UK population^11^. There is a marked discrepancy between the number of vCJD cases suggested by this figure and the relatively small number of people who have actually developed vCJD. This raises important questions about whether individuals with PrP^Sc^ deposits in lymphoid tissues may eventually develop vCJD after prolonged incubation periods, or remain asymptomatic throughout their natural lifespan (referred to as subclinical infection), and whether they pose a risk of onward disease transmission through blood or organ donation, or contamination of instruments during surgical procedures.

The potential for reservoirs of subclinical infection in a population also poses challenges for control of animal prion diseases, such as scrapie. Classical scrapie has been successfully controlled by selective breeding programmes, such as the National Scrapie Plan in the UK, that have encouraged breeding from sheep that are genetically resistant to the disease^12^. One criticism of such programmes was that they might eliminate clinical disease while allowing subclinical scrapie infection to persist undetected in resistant genotypes, perhaps leading to future disease outbreaks. No convincing evidence of this has yet emerged, but the factors that lead to subclinical prion infections have received relatively little research attention, and are not well understood. Limited studies in rodents have indicated that, in addition to cross-species transmission, low prion doses and specific immune deficiencies may also predispose to the development of subclinical infection^13^.

We have previously used sheep experimentally infected with BSE as a model to study the risks of transmission of prion infection by blood transfusion^14–16^. In these experiments, blood collected from donor sheep orally infected with BSE was separated into different components (red cell concentrate, plasma and platelets), with each component being transfused into an individual recipient sheep. Whilst some infected donors transmitted infection to some or all of their respective recipients, a large number of recipient sheep did not develop clinical disease^15,16^ during several years of follow-up, and showed no evidence of PrP^Sc^ in brain or lymphoid tissues using conventional post mortem tests (immunohistochemistry, Western blot). We hypothesized that since these sheep were most likely exposed to a low dose of BSE via transfusion, a proportion of them might be subclinically infected, with PrP^Sc^ levels below the threshold of detection for the conventional tests. To test this hypothesis, we screened lymphoid tissues (prescapular lymph node; PSLN) from clinically negative, pathologically negative recipient sheep for the presence of PrP^Sc^ using a highly sensitive protein misfolding cyclic amplification assay (PMCA). In a subset of animals with consistently positive PMCA results in the PSLN, we extended the PMCA analysis to other lymphoid tissues, brain and blood samples.

## Results

### Sensitivity of PMCA detection of PrP^Sc^ in lymph nodes from BSE-infected sheep

**Figure 1:**
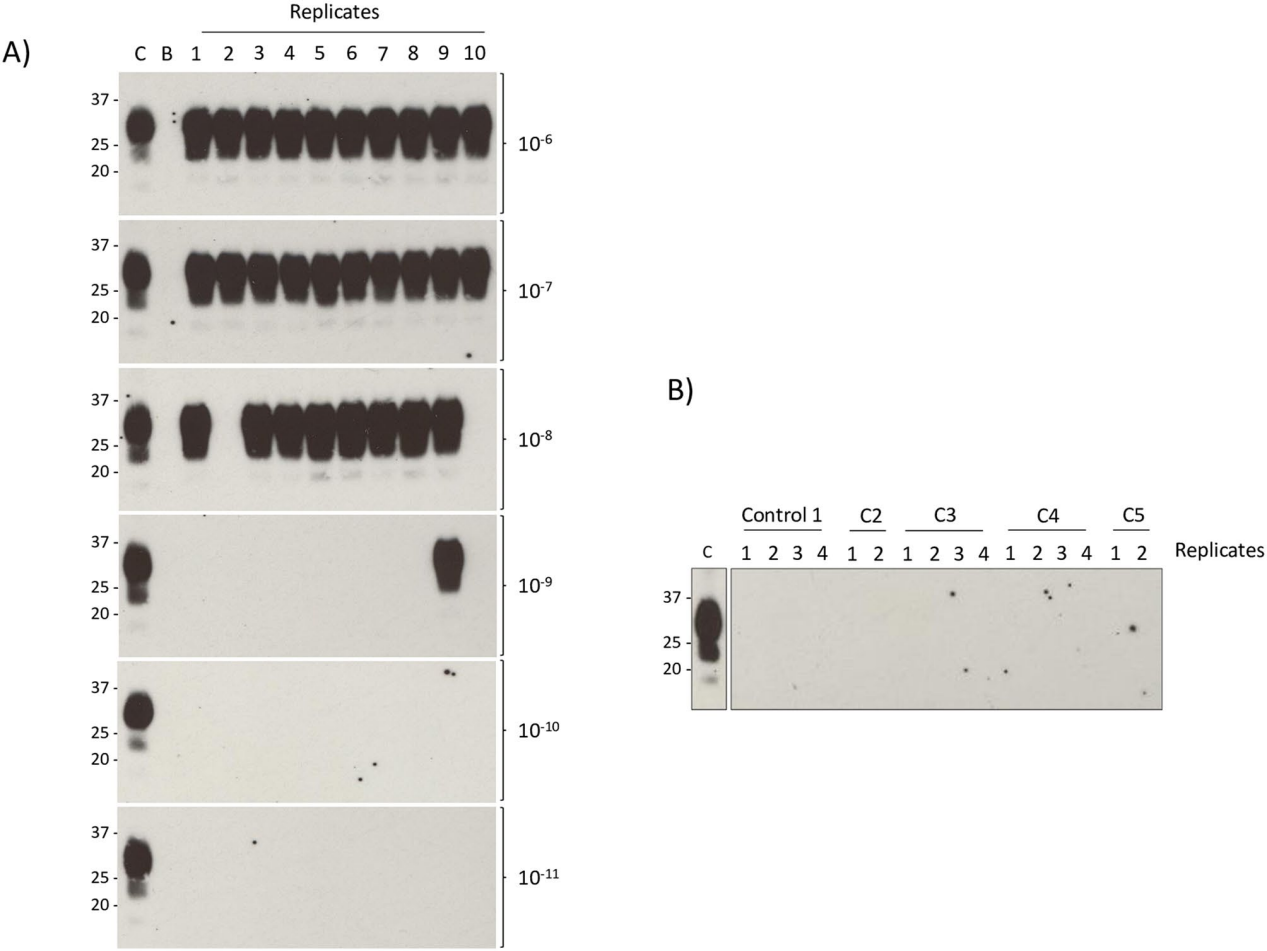
Limits of detection of PrP^Sc^ in prescapular lymph node from a pathology positive sheep. (**A**) Tenfold dilutions of PSLN homogenate (10^–6^ to 10^–11^) from a BSE-infected recipient sheep (M525) were used to seed PMCA reactions, with 10 replicates at each dilution. Following two rounds of PMCA, reaction products were tested for the presence of PK-resistant PrP^Sc^ by Western blotting. C—positive control; BSE-infected sheep brain homogenate (PK digested; 1.7 mg brain equivalent). B—blank lane. (**B**) Negative controls that were run in the same plate. Controls included: C1—substrate only, C2—substrate plus dextran, C3—prescapular lymph node from a mock-infected sheep (N170), C4—brain homogenate from the same mock-infected sheep (N170), C5—brain homogenate from another mock-infected sheep (P457). C—positive control—PK-treated unamplified brain homogenate from BSE-infected sheep (1.7 mg brain equivalent).

We reasoned that subclinical infection might be more readily detected by analysis of lymphoid tissues, rather than brain. Initially, we chose the prescapular (or superficial cervical) lymph node (PSLN), a large subcutaneous lymph node draining the skin and underlying muscles in the lower neck and thorax, as a target for screening. In order to test limit of detection of the PMCA assay in lymphoid tissues, tenfold serial dilutions of PSLN homogenate (with 10 replicate reactions per dilution) from a recipient sheep that had developed clinical signs of BSE and tested positive for PrP^Sc^ deposition in brain and lymphoid tissues by IHC and Western blot (designated “pathology positive”) were analysed by PMCA. As shown in Fig. 1, after two rounds of amplification, all replicates gave positive PMCA results to a dilution of 10^–7^, eight out of ten replicates were positive at 10^–8^, and only one replicate was positive at 10^–9^. Using the Spearmann–Karber method, the concentration of seeding units (SD_50_ = dilution at which 50% replicate PMCA reactions are positive) for this positive sample was calculated to be 5.02 × 10^10^ SD_50_ per gram of lymph node. Surprisingly, this is only one log less than the SD_50_ titre calculated for a BSE-infected sheep brain pool similarly titrated in PMCA (5.02 × 10^11^ SD_50_/g brain).

Previous data suggested that the *PRNP* codon 141 polymorphism (L141F), which is associated with statistically significant differences in incubation period in BSE-infected sheep^17^, might also influence the extent of PrP^Sc^ deposition in lymphoid tissues^15^. On average, 141LL sheep had the shortest incubation periods and the most widespread deposition of PrP^Sc^ in lymphoid tissue, whilst 141LF sheep had the longest survival periods and more restricted distribution of PrP^Sc^ in lymphoid tissues. To test whether the codon 141 polymorphism was associated with differences in PrP^Sc^ concentrations in the PSLN, we performed PMCA on serial dilutions (5 replicate reactions per dilution) of PSLN homogenate from three pathology positive recipients of each *PRNP* codon 141 genotype (141LL, 141FF and 141LF). For 141FF and 141LF genotypes, there was marked inter-individual variation in the calculated SD_50_ values (Table S1). The average SD_50_ values (± standard deviation) per gramme of lymph node were 1.7 ± 0.76 × 10^10^ SD_50_/g for 141LL, 6.5 ± 8.2 × 10^9^ SD_50_/g for 141FF, and 2.3 ± 3.5 × 10^9^ SD_50_/g for 141LF, and the differences between genotypes were not statistically significant when compared using student T tests. Although there does appear to be a trend towards higher PrP^Sc^ concentrations in lymphoid tissues of 141LL sheep, and lower concentrations in 141FF and 141LF animals, the large inter-individual variations in PSLN PrP^Sc^ concentrations most likely explain the lack of statistical significance. Thus, codon 141 genotype was not expected to have a significant effect on detection of PrP^Sc^ in lymphoid tissues from BSE-infected sheep.

### PrP^Sc^ detection in lymph nodes of sheep exposed to low doses of BSE by blood transfusion

To investigate the possibility of subclinical infection, we used PMCA to screen PSLN samples from sheep that had been transfused with components from BSE-infected donors, but survived for significant periods (up to 10–11 years) following transfusion, and did not show clinical signs of BSE or PrP^Sc^ deposition in brain and lymphoid tissues by standard post mortem tests (IHC and Western blot). For the purposes of this discussion, they are therefore designated “pathology negative”.

**Figure 2:**
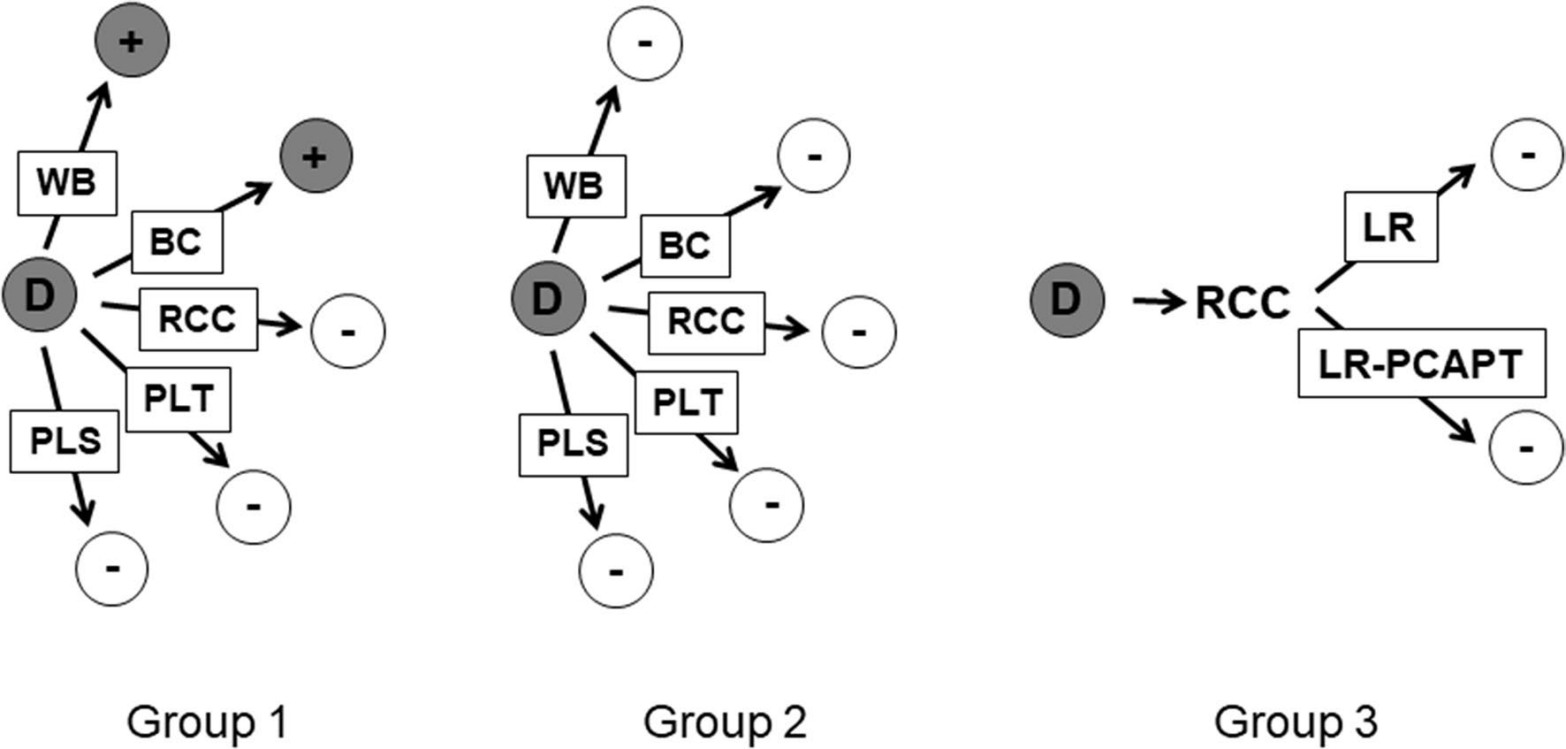
Schematic representation of sheep blood transfusion experiments**.** Whole blood (WB) was collected from BSE-infected donor sheep (D), processed into red cell concentrate (RCC), buffy coat (BC), plasma (PLS) and platelets (PLT), and each component transfused into an individual recipient sheep. In some cases, leucodepleted RCC, PLS, and PLT were also transfused, producing a cohort of 7 recipients per donor (WB was not transfused in these cases). Some recipients developed clinical signs of BSE and/or tested positive by Western blot/IHC (referred to as pathology positive; filled circles +), others did not develop clinical signs of BSE and/or tested negative by Western blot/IHC (referred to as pathology negative; open circles −). Group 1: some recipients were pathology positive. Group 2: no pathology positive recipients. Group 3: P-CAPT experiment: recipients transfused with leucodepleted (LR) or leucodepleted and P-CAPT filtered (LR-PCAPT) red cell concentrate (RCC). Details of the experimental design and results for all groups can be found in previous publications^15,16^.

We tested three distinct groups of sheep (Fig. 2):

- Group 1: Pathology negative sheep that formed part of a cohort of recipients of components from a single infected donor, in which one or more recipients became infected following transfusion (n = 21). The rationale was that the donor’s blood was known to be infectious at the time of transfusion, and therefore it is most likely that these sheep were exposed to low titres of infectivity.
- Group 2: Pathology negative sheep that formed part of a cohort of recipients of components from a single infected donor, in which none of the recipients became infected following transfusion (n = 30). We reasoned that these sheep might not have been exposed, or would have been exposed to lower doses of infection than in Group 1, as the donor’s blood was not infectious at the time of transfusion.
- Group 3: Pathology negative sheep (n = 10) from a separate experiment to determine the efficacy of prion filters (P-CAPT) in preventing transmission of BSE by transfusion^16^. In this study, blood was collected from donor sheep during the clinical phase of infection, and used to prepare paired units of red cells, one of which was leucodepleted (LR-RCC, and the other leucodepleted and filtered to remove prions (LR-PCAPT-RCC); each unit was transfused into a single recipient sheep. Since donor sheep were all showing clinical signs of BSE, we would expect that their blood would be highly infectious at this point, based on data from previous studies^14^. Therefore it is probable that low levels of infection would remain after leucodepletion and prion filtration steps.

A sample of prescapular lymph node (PSLN) from each sheep was homogenised and used to seed PMCA reactions (in duplicate or triplicate). Positive controls consisted of BSE-infected sheep brain homogenate and homogenates of PSLN from two recipient sheep confirmed as BSE positive by IHC and Western blot. Negative controls were PSLN homogenates from negative control mock-infected donors (n = 5) and recipients (n = 5). Samples from Groups 1 and 3 were run in an initial series of experiments (Series 1); Group 2 samples were tested in a second series of experiments (Series 2). The results of testing in the three groups of recipients are summarized in Table 1 (see Supplementary Information; Table S2 for the full results from individual animals). Each sample was tested in at least 3 repeat PMCA experiments, since the majority of samples producing positive PMCA results showed variability in the numbers of positive replicates between experiments. This suggests that the PrP^Sc^ levels in these samples were close to the limit of detection of the assay, equivalent to that in a 10^–6^-10^–9^ dilution of PSLN from a pathology positive animal (i.e. at least a million-fold lower than that in pathology positive animals; see Fig. 1, Table S1).

**Table 1:** Summary of PMCA test results on prescapular lymph node samples from “pathology negative” sheep.

Group	Survival period (days post-infection)	No. repeat PMCA experiments	No. animals with at least 1 positive PMCA result	No. animals with negative PMCA results	Percentage of positive replicates for all experiments (median)
Group 1 (n = 21)	333–4000	3–10	18	3	0–100% (21%)
Group 2 (n = 30)	400–3995	3–4	16	14	0–100% (11%)
Group 3 (n = 10)	573–3476	3–6	6	4	0–89% (25%)
Positive controls (n = 2)	468, 609	5–10	2	0	100%
Negative controls (n = 10)—series 1^a^	312–3434	7–10	1	9	0–6% (0%)
Negative controls (n = 10)—series 2^b^	312–3434	4–9	2	8	0–16% (0%)

^a^Series 1—negative controls run in experiments with test samples for Groups 1 and 3.

^b^Series 2—negative controls run in experiments with test samples for Group 2.

In Group 1, the majority of samples (18/21) tested positive in at least one PMCA experiment. The PMCA positive samples came from recipients transfused with buffy coat (n = 1), red cells (n = 5), platelets (n = 5), leucodepleted platelets (n = 1), plasma (n = 5) and leucodepleted plasma (n = 1). The three samples that tested negative by PMCA were all from sheep transfused with plasma. Only one animal (P241) gave positive PMCA results for all 3 replicates in repeat experiments (Table S2), similar to positive control samples from pathology positive sheep. This sheep was culled at 333 days post transfusion, which is before the earliest onset of clinical signs in pathology positive recipients (391 days post transfusion), raising the possibility that this represents preclinical rather than subclinical infection. However, the rest of the sheep in this group lived for at least 1911 days post transfusion, i.e. significantly longer than the longest survival period (1323 days post transfusion) recorded for a pathology positive recipient. For the other animals giving positive PMCA results, there was marked inter-individual variation in the percentage of positive replicates across all experiments, ranging from 11 to 67%.

In Group 2, just over half the samples tested (16/30) gave a positive result in at least one PMCA experiment. The sheep with positive PMCA results were transfused with whole blood (n = 2), buffy coat (n = 10) or platelets (n = 4), and those with PMCA negative results had received whole blood (n = 2), buffy coat (n = 6), platelets (n = 4) or red cells (n = 2). Again, the percentage of positive replicates across all experiments differed markedly between individuals, ranging from 11 to 100%, with only one animal (P353) giving positive results for all 3 replicates in every experiment. The survival time for this recipient was 2109 days post transfusion i.e. well beyond the expected range of survival periods for clinically affected sheep.

In Group 3 (pathology negative sheep from the P-CAPT study), over half the samples (6/10) also gave a positive result in one or more repeat PMCA experiments. The sheep with positive PMCA results were transfused with leucodepleted red cells (n = 3) or leucodepleted and P-CAPT filtered red cells (n = 3). For these six samples, the percentage of positive replicates across all PMCA experiments ranged from 6 to 89%.

To check for the possibility of non-specific amplification by PMCA, we ran a panel of ten negative control PSLN samples from mock-infected sheep in each series of experiments. Positive PMCA results were observed for two negative control samples in some experiments (Table 1; Table S2). This was judged to be more likely due to contamination of the seed homogenates rather than non-specific amplification because it occurred in samples from only two individual sheep, rather than randomly among all negative controls, and following preparation of fresh seed homogenates from the original tissue samples, PMCA reactions were consistently negative.

Analysis of the data using generalized linear mixed models (GLMMs) revealed statistically significant differences between the PMCA results from each of Groups 1–3 and the negative controls, with p values of < 0.001 for Groups 1 and 3, and 0.02 for Group 2. There was no statistically significant difference in the results for Group 1 and Group 3. Although a lower proportion of animals in Group 2 gave positive PMCA results compared to Group 1, the difference was not quite statistically significant (p = 0.054).

### PMCA analysis of other tissues in pathology negative sheep

Our initial PMCA analysis of PSLN from pathology negative sheep showed that a relatively high proportion of animals gave positive results, but suggested that the PrP^Sc^ concentrations were much lower than those found in the PSLN of BSE-infected sheep. The next step was to determine whether we could detect PrP^Sc^ in other tissues and blood samples from these sheep. We selected one sheep from Group 1 (P241), four sheep from Group 2 (P225, P243, P248, P353) and three sheep from Group 3 (P467, P304, Q397), which gave the most consistent positive results for PSLN in repeated PMCA experiments (50–100% of positive replicates). All eight sheep were heterozygous at *PRNP* codon 141 (141 LF). Samples of other lymphoid tissues, brain and blood (buffy coat) from these sheep were prepared and used to seed PMCA reactions (Table 2).

**Table 2:** PMCA results and limit of detection in tissues from selected sheep with positive PMCA in PSLN.

Group	Sheep ID	Component transfused	Survival period (days post infection)	Tissues tested No. replicates positive/No. tested (Limit of detection*)						
				Prescapular LN	Mesenteric LN	Peyer’s Patch	Tonsil	Spleen	Brain	Buffy coat
1	P241	Red cells	333	3/3 (10^–4^)	0/3 (NA)	NT	3/3 (NT)	3/3 (10^–3^)	3/3 (10^–3^)	0/3 (NA)
2	P225	Buffy coat	503	3/3 (10^–1^)	0/2 (NA)	2/2 (10^–2^)	2/2 (10^–1^)	0/2 (NA)	0/2 (NA)	0/2 (NA)
	P243	Buffy coat	2323	3/3 (10^–2^)	0/2 (NA)	0/2 (NA)	0/2 (NA)	0/2 (NA)	0/2 (NA)	0/2 (NA)
	P248	Whole blood	2102	3/3 (10^–1^)	0/2 (NA)	0/2 (NA)	0/2 (NA)	0/2 (NA)	0/2 (NA)	0/2 (NA)
	P353	Buffy coat	2109	3/3 (10^–1^)	0/2 (NA)	0/2 (NA)	0/2 (NA)	0/2 (NA)	0/2 (NA)	0/2 (NA)
3	P467	LR-RCC	573	3/3 (10^–1^)	2/3 (10^–1^)	NT	NT	3/3 (10^–2^)	3/3 (10^–2^)	0/3 (NA)
	P304	LR-PCAPT-RCC	2220	0/3 (NT)†	NT	NT	3/3 (10^–2^)	3/3 (10^–2^)	3/3 (10^–3^)	0/3 (NA)
	Q397	LR-PCAPT-RCC	3476	3/3 (10^–1^)	0/3 (NA)	0/3 (NA)	0/3 (NA)	0/3 (NA)	0/3 (NA)	0/3 (NA)

*NA* not applicable, *NT* not tested.

*Highest dilution of tissue homogenate giving positive PMCA result.

^†^Positive in several other experiments for 10^–1^ dilution (see Table S2).

In three sheep (P241, P467, P304), at least two other lymphoid tissues e.g. spleen, mesenteric lymph node, tonsil, as well as brain samples also gave positive results in PMCA, but buffy coat samples were negative. One sheep (P225) gave positive PMCA reactions for tonsil and ileal Peyer’s patch samples, but not for brain or buffy coat. The remaining four sheep (P243, P248, P353, Q397) gave negative results in PMCA for all samples apart from PSLN.

Three of the sheep (P241, P467 and P225) were culled due to intercurrent disease at relatively short intervals post transfusion (333, 573 and 503 days post-infection respectively) (Table S2). All three sheep had the *PRNP* genotype 141LF and, for comparison, survival periods of clinically positive, pathology positive 141LF recipients ranged from 658 to 1323 days post-infection (mean ± SD = 1025 ± 189 days). It could therefore be argued that P241, P467 and P225 may have been in the preclinical phase of infection at the time of death, and would have progressed to clinical disease if they had lived longer. The other five sheep survived for at least 2102 days (> 2 years longer than the maximum survival time in pathology positive recipients), but only one (P304) had detectable PrP^Sc^ in tissues other than the PSLN.

PMCA analysis of serial tenfold dilutions of samples that tested positive showed that the limits of detection were reached at dilutions of 10^–1^–10^–4^ (Table 2). In comparison, with tissues from pathology positive sheep, the limits of detection were reached at dilutions of 10^–9^–10^–10^ for brain^15^ and 10^–6^–10^–9^ for PSLN (Fig. 1; Table S1). This result provides additional evidence that the PrP^Sc^ concentrations in the tissues of the pathology negative sheep are several orders of magnitude lower than those typically found in tissues of pathology positive sheep.

### PMCA analysis of lymphoid tissues from secondary recipients

Based on our previously published data^15^, we reasoned that since blood samples from pathology negative subclinical recipient sheep tested negative by PMCA, they were unlikely to transmit BSE by blood transfusion. In order to test this, we carried out PMCA analysis on PSLN and brain samples from ten pathology negative secondary recipients. These sheep were transfused with whole blood collected from primary recipients at 15 months post transfusion. In six of the primary recipients, PrP^Sc^ was detected in the PSLN by PMCA, but the samples from all secondary recipients tested negative in PMCA (Table S3). This result shows that subclinically infected sheep were not able to transmit BSE via blood transfusion at this stage of infection.

### Detailed immunohistochemical analysis of tissue samples from pathology negative sheep

**Figure 3:**
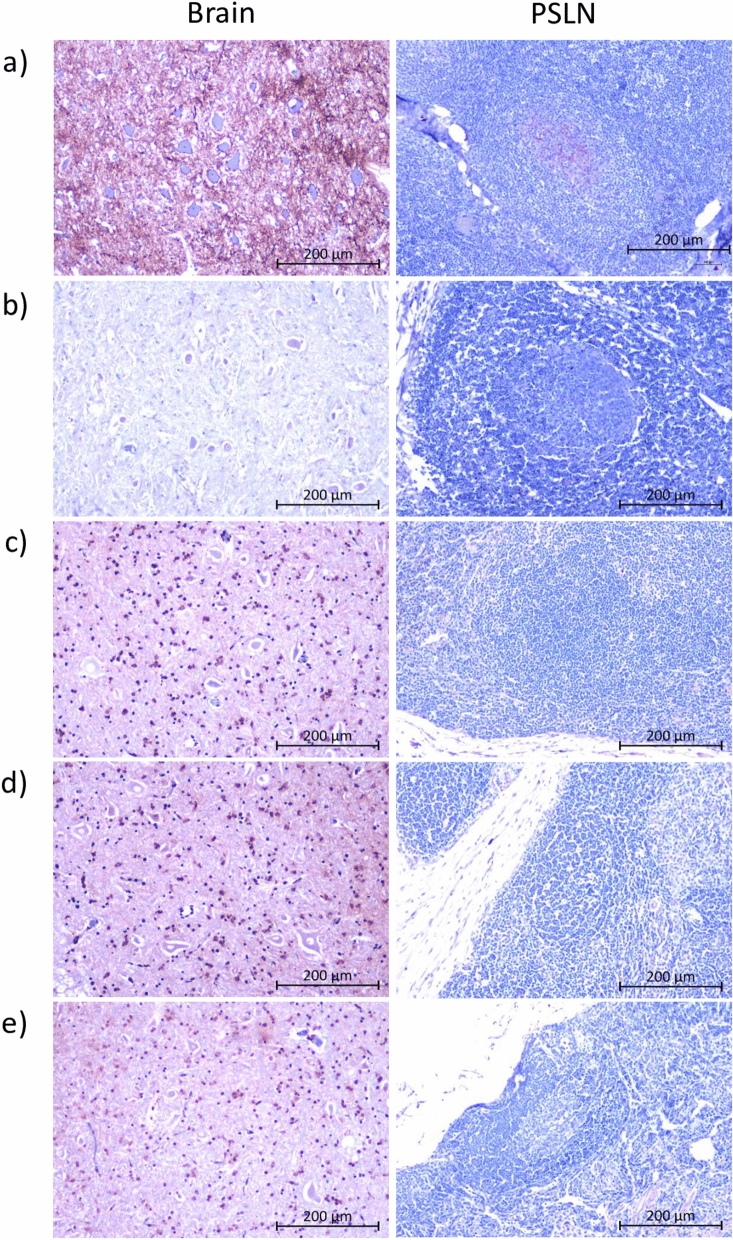
Immunohistochemical labelling of multiple sections of PSLN and brain from pathology negative sheep for PrP^Sc^. Sections of PSLN and brain medulla (at the level of the obex) were cut from the same block at five different levels 50 µm apart, and IHC performed with monoclonal antibody BG4. Representative results from one sheep (Q397) are shown. (**a**) Positive control sections from BSE-infected pathology positive sheep; (**b**) negative control sections from mock-infected sheep; (**c**–**e**) three sections cut at least 50 µm apart. Scale bars = 200 µm.

Surveys of human appendix and tonsil samples using immunohistochemistry (IHC) detected a prevalence of abnormal PrP deposition that was inconsistent with the observed numbers of clinical vCJD cases, suggesting that there may be a significant level of subclinical vCJD infection in the UK population^11^. To determine whether our standard IHC procedures (on a single tissue section) may have missed small PrP^Sc^ deposits in PMCA positive tissues, we cut additional sections from the PSLN and obex (medulla oblongata) blocks of four pathology negative sheep (P241, P467, P304, Q397). Sections were cut at five different levels 50 μm apart, and IHC was performed using the monoclonal antibody BG4. None of the additional sections showed positive labelling (Fig. 3).

## Discussion

The incidence and importance of subclinical animal and human prion disease in transmission of infection is not well understood, due to challenges in identifying and monitoring infected individuals throughout their lifespan. In addition, the prolonged incubation periods of prion diseases can make it difficult to distinguish subclinical from preclinical infection. In this study, we have shown that a high proportion of sheep transfused with blood components from BSE-infected preclinical donors may survive for more than 10 years (i.e. close to their expected lifespan) without developing clinical signs of disease, yet harbour PrP^Sc^ in lymph nodes that is detectable using a sensitive amplification assay (PMCA). For the majority of these sheep this most likely represents true subclinical infection, although we cannot exclude the possibility that some were preclinical (due to relatively short survival times). PMCA analysis of other lymphoid tissues, brain and blood (buffy coat) samples from a small subset of these animals demonstrated variability in the tissues containing PrP^Sc^, although all buffy coat samples were negative. Titration experiments demonstrated that the PrP^Sc^ concentrations in PMCA positive lymphoid tissues from pathology negative sheep were at least 100,000 times lower than equivalent tissues from pathology positive sheep.

Our previous study showed that the highest levels of infectivity were associated with whole blood and buffy coat, followed by platelets, red cells and plasma, in that order. Leucodepletion resulted in a substantial and significant reduction in infectivity in platelets, red cells and plasma, but did not completely prevent transmission of infection by transfusion. In this study, subclinical infection was found in recipients of whole blood, buffy coat, platelets, red cells and leucodepleted platelets and plasma, but the numbers of sheep analysed did not allow for statistical analysis of association of subclinical infection with specific blood components. In the P-CAPT study, the additional prion filtration step did not appear to have any effect on the establishment of subclinical infection, as equal numbers of pathology negative recipients of leucodepleted or leucodepleted/prion filtered RCC tested positive by PMCA.

In experimental models, subclinical infection has been studied most often in the context of cross-species transmission of prions, where the species transmission barrier contributes to higher rates of subclinical infection in primary and secondary passages^8,9^. Other factors that may contribute to the development of subclinical rather than clinical infection include the dose and route of infection^13,18^, and the recipient’s genotype, age and immune status^19–21^. In our study, there was no transmission barrier, and recipient sheep were young healthy adults (< 2 years old) with very similar *PRNP* genotypes, apart from the codon 141 polymorphism, which despite its association with survival period does not appear to influence susceptibility to infection with BSE. Therefore, it is likely that subclinical infection in this model is the result of exposure to low doses of infectivity, and/or the route and method of infection (intravenous blood transfusion).

Most experimental infections with prions are performed by intracerebral, intraperitoneal or oral infection with infected brain homogenates, and end-point titrations are frequently used to estimate the minimal infectious dose or ID_50_. The biochemical nature of blood-borne prions may differ significantly from those derived from brain tissue, and previous experiments titrating scrapie-infected blood components by transfusion in sheep estimated that the minimal infectious dose for blood was approximately 1000 times lower than for brain^22^. In some of the latter experiments, a few recipient sheep transfused with lower dose components did not develop clinical disease within the timeframe of the experiment (400–450 days), but showed post mortem evidence of PrP^Sc^ deposits in lymphoid and brain tissues by Western blot and/or IHC^22^. This would be consistent with subclinical infection of these sheep, and is similar to our findings, although by using the more sensitive PMCA technique we were able to detect PrP^Sc^ in a higher proportion of clinically negative recipients. Subclinical infection has also been reported in one bovine inoculated intracerebrally with BSE^23^, in five goats orally infected with L-BSE^24^, and in five white-tailed deer orally infected with urine and faeces from CWD-positive animals^25^. In common with our findings, these studies reported that standard post mortems tests (IHC, Western blot, ELISA) performed on tissues were negative, and PrP^Sc^ was detected only following amplification by PMCA or the related real time quaking induced conversion assay (RT-QuIC). The range of tissues that tested positive varied across studies, most likely reflecting differences in the experimental models, prion strains, routes of infection, and the period of follow up after infection. In this study, we found that PrP^Sc^ was detected most consistently in the lymphoid tissues (particularly the prescapular lymph node) of subclinical sheep by PMCA, with four out the eight sheep in which multiple tissues were tested having detectable PrP^Sc^ in PSLN only.

It is unclear why the prescapular lymph node should be a preferred site for persistence of prion infection. IHC results from pathology positive recipients revealed widespread PrP^Sc^ deposition in the lymphoid tissues examined (PSLN, ileal Peyer’s patch, mesenteric lymph node, spleen, tonsil), with the most consistent detection in Peyer’s patch, tonsil and spleen^15^. The four sheep that tested PMCA positive in PSLN only were more than 6 years old at the time they were culled (the oldest was > 10 years old), and it is possible that involution of certain lymphoid tissues, particularly ileal Peyer’s patch, with increasing age may partly explain the inability to detect PrP^Sc^ in these samples.

Three of the eight sheep in which multiple tissues were tested by PMCA showed evidence of PrP^Sc^ deposition in the brain, which indicates that neuroinvasion has occurred and might be expected to lead to clinical disease. Two of these sheep (one from Group 1 and one from Group 3) had survival periods that were less than the shortest survival time of clinically positive sheep of the same *PRNP* genotype (141LF), and it is therefore possible that they were in the preclinical stages of infection. However, the third sheep (P304), also from Group 3, survived more than 6 years post transfusion without developing clinical signs of BSE, which is more consistent with true subclinical infection.

Important issues arising from our findings are whether subclinically infected individuals are capable of transmitting infection, or whether certain events or processes might be able to trigger clinical disease. We have shown that the levels of PrP^Sc^ found in the tissues of subclinically infected sheep are at least 100,000 times less on average than those in clinically affected animals, and that PrP^Sc^ was not detectable in blood samples. It is known that tissue PrP^Sc^ concentrations do not always accurately reflect infectivity titres^26–28^, although recent studies in a variety of models have found some correlation between infectivity and PrP^Sc^ seeding activity measured by PMCA or RT-QuIC^29–31^. This relationship needs to be established for each model system^32^, so that bioassays of PMCA positive tissues would be required to make accurate predictions about infection risks associated with subclinical BSE infection in sheep. However, our previous work in the same sheep showed that blood samples from BSE-infected sheep that tested negative in PMCA were much less likely to transmit infection by transfusion than PMCA positive blood samples^15^. Since the blood samples from subclinical sheep consistently tested negative in PMCA, this would suggest that they pose a low risk of transfusion transmission, and possibly also transmission via body fluids. The limited results from secondary recipients provide support to the hypothesis that subclinical sheep are not infectious, since their tissues tested negative by PMCA even if the respective donors were PMCA positive.

The persistence of low level infection over prolonged periods, without progression to clinical disease, implies a balance between mechanisms of prion clearance and replication. Even if subclinical individuals are not directly infectious/contagious, this long term persistence of infection might allow for selection of prion variants or strains that can escape host control mechanisms and transmit infection or cause disease. It is also possible that age-related declines in host homeostatic mechanisms, or events such as co-infection with other pathogens, might result in increased prion replication and the late appearance of clinical disease. Whilst these are currently hypothetical possibilities, there is accumulating evidence from experimental models that systemic inflammation or co-infection with various pathogens can accelerate prion pathogenesis^33^.

Another important question is whether our observation of apparent subclinical infection in sheep is analogous to the detection of abnormal PrP deposits in anonymised human appendix samples^11^. Sheep do not have an anatomical structure equivalent to the human appendix, but we detected PrP^Sc^ in gut-associated lymphoid tissues (ileal Peyer’s patch) from one out of four sheep tested. In contrast to the appendix surveys, we found that PrP^Sc^ in lymphoid tissues and brain of subclinical sheep was not detectable by IHC, but only following amplification in PMCA, suggesting that levels of PrP^Sc^ were lower in sheep compared to human tissues. Even following more detailed examination of sections cut at different levels in the sheep samples (Fig. 3), we were unable detect any deposits of PrP^Sc^ by IHC. However, our results indicate that it is possible for subclinical infection to occur and persist over many years in a substantial proportion of individuals exposed to low prion doses by blood transfusion. It would be interesting to establish whether a similar prevalence of subclinical infection is found in sheep orally infected with very low doses of BSE, as many more people are likely to have been exposed to infection though diet than by transfusion.

Our study demonstrates for the first time that subclinical infection is a common outcome following low dose exposure to prions by a clinically relevant transmission route (blood transfusion), and can persist in hosts until close to their natural lifespan. By analogy, it is plausible that subclinical vCJD may occur in human patients who have been similarly exposed, and this could account for some of the IHC positive appendix samples identified in previous surveys. Importantly, we found that PrP^Sc^ amplification was essential for detecting subclinical infections in our study, supporting the case for targeted use of techniques such as PMCA and RT-QuIC in surveillance, to estimate prevalence of infection. Further research using animal models is vital to establish infection transmission risks associated with subclinical infection, and the role of subclinical infection in maintenance and re-emergence of prion diseases in animal and human populations.

## Materials and methods

### Ethics statement

This study was reviewed and approved by animal welfare and ethics review committees at the Institute for Animal Health (Compton) and The Roslin Institute, and carried out under the authority of Home Office Project Licences (references: 30/2282, 60/4143, 70/8595), and in accordance with ARRIVE guidelines.

### Experimental infection of sheep

Cheviot sheep were sourced from the Defra scrapie-free flock, which was derived from sheep imported from New Zealand^34^. All sheep had the *PRNP* genotype ARQ/ARQ (using standard terminology for amino acids encoded by codons 136, 154 and 171 of the *PRNP* gene), which has the shortest incubation period and a high attack rate when experimentally infected with BSE^35^. They did not have additional polymorphisms at codons 112 and 168 (M112T and P168L, respectively) that have been associated with reduced susceptibility to prion disease in sheep^36,37^. However, they did have relatively high frequencies of the L141F polymorphism at codon 141, which at the time the experiments were initiated (2006) was not known to influence susceptibility to BSE^17^.

The experimental design for the main transfusion experiment is summarized in Fig. 2, and has previously been described in detail^15,38^. Briefly, donor sheep (n = 39) aged between 6 and 12 months were experimentally infected with BSE by administration of an oral dose of 5 g of BSE-infected cattle brain homogenate. Negative control sheep (n = 10) were orally dosed with 5 g of uninfected cattle brain homogenate. At 10 months post-infection, two units of whole blood (WB; 1 unit = 450 ml ± 10% v/v) were collected from each infected donor, and processed to yield red cell concentrate (RCC), platelets (PLT), plasma (PLS), each with or without leucodepletion, and buffy coat (BC), as previously described^38^. One unit of whole blood was collected from negative controls but not processed into components. Recipient sheep aged between 6 and 24 months were transfused with blood components prepared from infected and negative control donors. Eighteen recipients (transfused with either whole blood or buffy coat from orally infected donors) were used as donors of whole blood (1 unit = 450 ml ± 10% v/v) for transfusion into secondary recipients^38^.

The experimental design of the P-CAPT study (Fig. 2) has also been previously described^16^. Briefly, two units of whole blood were collected from seven donor sheep orally infected with BSE and showing clinical signs of disease, immediately before they were euthanized. Both units of blood were processed to prepare leucodepleted RCC (LR-RCC), and one unit was further passed through a prion removal filter (P-CAPT; MacoPharma) (LR-PCAPT-RCC). Each component was transfused into an individual recipient sheep (14 recipients in total).

Procedures for housing, management and clinical scoring of sheep are as previously described^38^. Sheep with positive TSE clinical scores were euthanized once they reached defined humane end points. Other sheep were euthanized because of intercurrent disease or welfare concerns, or at the end of different phases of the project. Throughout infection, samples of blood were collected from a subset of donors and recipients, fractionated into plasma, buffy coat and red cells, and archived at − 80 °C. Samples of brain, palatine tonsil, spleen, ileal Peyer’s patch, mesenteric lymph node and prescapular lymph node were collected at necropsy and fixed in neutral buffered formalin, or frozen at − 80 °C, as previously described. To confirm infection with BSE, tissue samples were analysed by immunohistochemistry (IHC) and Western blotting for detection of disease-associated PrP^Sc^, as previously described^39^. For all sheep, the survival period (SP) was calculated as the interval between the date of infection and the date of euthanasia. Detailed tables of the clinical scores, survival periods, and results of IHC and Western blotting for all donor and recipient sheep in the leucodepletion experiment have been published^15^.

### Protein misfolding cyclic amplification for detection of PrP^Sc^

We used a microplate-based, miniaturized bead serial PMCA protocol^15,40,41^. Briefly, brain homogenate from TgshpXI transgenic mice over-expressing sheep PrP^C^ from the ARQ *PRNP* allele was used as substrate. The mouse brains were homogenized in PMCA buffer (PBS pH 7.2, 0.25% v/v Triton X100, 150 mM NaCl, Roche cOmplete Protease Inhibitor) at 4 °C to provide a 10% w/v homogenate, using a pestle and mortar (Dounce) glass homogenizer. Samples of tissues from BSE-infected sheep and controls were dissected, weighed and homogenized in PMCA buffer using an Omni electronic homogeniser with disposable probes (Camlab), to yield 10% w/v homogenates (termed “seed”). The substrate and seed homogenates were passed through 25 G needles, then divided into smaller aliquots and stored at − 80 °C. Sheep blood samples used as “seed” were not homogenized, but were thawed from − 80 °C, and used undiluted.

PMCA reactions were performed in Axygen 96 well PCR microtitre plates, with 45 µl of substrate homogenate and one Teflon bead (2.381 mm, Marteau & Lemarie, France) per well, seeded with 5 µl seed homogenate or blood sample. 5% w/v dextran sulphate (Sigma-Aldrich) solution was added to each reaction to give a final concentration of 0.5%. Positive controls run in each plate consisted of tenfold serial dilutions of PSLN homogenate from a BSE positive sheep, and tenfold serial dilutions of pooled brain homogenate from five BSE positive sheep, as previously described^15^. Negative controls consisted of reactions seeded with tissue homogenates or blood samples from negative control (mock-infected) sheep, substrate only, and/or substrate with added dextran sulphate (to rule out non-specific amplification in the presence of dextran).

The microplate based PMCA reactions were performed in a microplate horn attached to a programmable Misonix Q700 sonicator (QSonica), supplied with a water circulation system to maintain temperature at 37 °C, as previously described^15^. Each PMCA round consisted of 96 cycles (24 h) of 10 s sonication (at an amplitude of 50–70%) and 14 min 50 s incubation. After each round of amplification, 5 µl reaction product from each well was mixed with 45 µl fresh substrate in a new microtitre plate, and PMCA amplification repeated. Aliquots of reaction products from each PMCA round were digested with 0.1 mg/ml proteinase-K (Qiagen) for 1 h at 37 °C, and PrP^Sc^ detected by Western blotting, as previously described^15^, using ROS-BC6 monoclonal antibody as the primary antibody at a concentration of 0.25 μg/ml.

### Statistical analyses

PMCA results were analysed using generalized linear mixed models (GLMMs) with a logistic link function. Separate models were fitted to analyse the two series of experiments. Series 1 included data for Group 1 (infected cohort), Group 3 (PCAPT) and negative controls, and Series 2 included data for Group 2 (uninfected cohort) and negative controls. Both models fitted group as a fixed effect, and animal ID and experiment ID as random effects.

SD_50_ values per gramme of tissue were compared between sheep with different *PRNP* codon 141 genotypes using Student T tests (two-tailed; heteroscedastic).

## Supplementary Information

Supplementary Information.

## Data Availability

All data generated or analysed during this study are included in this published article (and its Supplementary Information files) and in a previous publication (https://doi.org/10.1371/journal.ppat.1009276). Additional data from individual experiments are available from the corresponding author on reasonable request.
