# Supplementary material for: Subclinical infection occurs frequently following low dose exposure to prions by blood transfusion
Source: Sci Rep. 2022 Jun 28;12:10923. doi: 10.1038/s41598-022-15105-w (PMC9240018; doi:10.1038/s41598-022-15105-w)

Title: Subclinical infection occurs frequently following low dose exposure to prions by blood transfusion.

Authors:

M Khalid F Salamat[1], Paula Stewart[1], Helen Brown[1], Kyle BC Tan[1], Allister Smith[1], Christopher de Wolf[1], A Richard Alejo Blanco[1], Marc Turner[2], Jean C Manson[1], Sandra McCutcheon[1] and E Fiona Houston[1]*

Affiliations:

[1]The Roslin Institute, Royal (Dick) School of Veterinary Studies, University of Edinburgh, Easter Bush, Midlothian, Edinburgh

[2]Scottish National Blood Transfusion Service (SNBTS), The Jack Copland Centre, Edinburgh

*Corresponding author (fiona.houston@roslin.ed.ac.uk)

Table S1. Relationship between *PRNP* codon 141 genotype and PrP^{Sc} concentration in PSLN of BSE infected sheep.

Codon 141 genotype →	LL			FF			LF		
Sheep ID →	M525	P480	N237	M455	M237	N160	N490	P341	P445
	Number of positive PMCA replicates/number of replicates								
PSLN Dilution									
10^{-4}	NT	NT	NT	5/5	NT	5/5	NT	5/5	5/5
10^{-5}	NT	NT	NT	5/5	NT	5/5	NT	5/5	5/5
10^{-6}	NT	NT	NT	4/5	NT	5/5	NT	4/5	3/5
10^{-7}	5/5	5/5	5/5	1/5	5/5	3/5	5/5	0/5	0/5
10^{-8}	3/5	1/5	2/5	1/5	1/5	0/5	0/5	0/5	0/5
10^{-9}	0/5	0/5	0/5	0/5	1/5	0/5	0/5	0/5	0/5
10^{-10}	0/5	0/5	0/5	0/5	0/5	0/5	0/5	0/5	0/5
10^{-11}	0/5	0/5	0/5	0/5	0/5	0/5	0/5	0/5	0/5
log10 50% end point =	-8.1	-7.7	-7.9	-6.7	-7.9	-7.1	-7.5	-6.3	-6.1
50% end point =	$10^{-8.1}$	$10^{-7.7}$	$10^{-7.9}$	$10^{-6.7}$	$10^{-7.9}$	$10^{-7.1}$	$10^{-7.5}$	$10^{-6.3}$	$10^{-6.1}$
SD_{50} per 0.005g LN =	1.26E+08	5.01E+07	7.94E+07	5.01E+06	7.94E+07	1.26E+07	3.16E+07	2.00E+06	1.26E+06
SD50/g LN =	2.52E+10	1.00E+10	1.59E+10	1.00E+09	1.59E+10	2.52E+09	6.32E+09	3.99E+08	2.52E+08
Mean	1.70E+10			6.47E+09			2.33E+09		
Stndard deviation	7.64E+09			8.19E+09			3.46E+09		

Key: NT Not tested

Table S2. Results of PMCA analysis of prescapular lymph node (PSLN) samples from individual pathology negative sheep.

Sheep ID	Status	Codon 141 *PRNP* genotype	Route of infection	Component transfused	Survival period (days post-infection)	No. of PMCA experiments	Total no. of replicates	No. of positive replicates	% positive replicates
P480	Positive control	LL	Transfusion	WB	468	10	30	30	100
M237	Positive control	FF	Transfusion	PLT	609	5	14	14	100
**Negative controls - Experiment series 1**									
N239	Negative control	LL	Oral	NA	2014	9	22	0	0
N170	Negative control	FF	Oral	NA	1785	10	24	1	4
N256	Negative control	LF	Oral	NA	2014	9	22	0	0
N217	Negative control	FF	Oral	NA	1785	8	20	0	0
N152	Negative control	FF	Oral	NA	1747	7	17	0	0
M308	Negative control	LL	Transfusion	WB	2142	4	11	0	0
P260	Negative control	LF	Transfusion	WB	3434	7	13	0	0
P194	Negative control	FF	Transfusion	WB	1364	7	17	0	0
P423	Negative control	LF	Transfusion	WB	312	7	17	0	0
P457	Negative control	LF	Transfusion	WB	2402	7	17	0	0
**Negative controls - Experiment series 2**									
N239	Negative control	LL	Oral	NA	2014	7	19	0	0
N170	Negative control	FF	Oral	NA	1785	9	43	7	16
N256	Negative control	LF	Oral	NA	2014	5	15	0	0
N217	Negative control	FF	Oral	NA	1785	5	15	0	0
N152	Negative control	FF	Oral	NA	1747	5	15	0	0
M308	Negative control	LL	Transfusion	WB	2142	5	15	0	0
P260	Negative control	LF	Transfusion	WB	3434	5	15	0	0
P194	Negative control	FF	Transfusion	WB	1364	4	12	0	0
P423	Negative control	LF	Transfusion	WB	312	8	33	2	6
P457	Negative control	LF	Transfusion	WB	2402	4	12	0	0

Sheep ID	Status	Codon 141 *PRNP* genotype	Route of infection	Component transfused	Survival period (days post-infection)	No. of PMCA experiments	Total no. of replicates	No. of positive replicates	% positive replicates
**Group 1**									
P545	Test animal	LF	Transfusion	PLS	3982	4	11	2	18
M216	Test animal	LL	Transfusion	RCC	1911	5	14	3	21
N255	Test animal	LF	Transfusion	PLT	2356	5	14	6	43
M546	Test animal	FF	Transfusion	RCC	2178	10	29	12	41
M226	Test animal	FF	Transfusion	PLS	2178	7	20	4	20
P241	Test animal	LF	Transfusion	RCC	333	6	18	18	100
P561	Test animal	LF	Transfusion	PLT	3948	9	27	12	44
P483	Test animal	LF	Transfusion	PLT	3961	3	9	3	33
P429	Test animal	LF	Transfusion	PLS-LR	4000	3	9	1	11
M527	Test animal	LL	Transfusion	RCC	2287	4	12	3	25
P204	Test animal	FF	Transfusion	PLS	2308	6	18	4	22
P515	Test animal	FF	Transfusion	PLT	3898	6	18	2	11
P463	Test animal	FF	Transfusion	PLT-LR	2936	4	12	8	67
P542	Test animal	LF	Transfusion	BC	3829	5	15	9	60
M524	Test animal	LF	Transfusion	PLS	2493	3	9	0	0
N325	Test animal	FF	Transfusion	PLS	2183	3	9	0	0
M497	Test animal	LF	Transfusion	PLS	2288	3	9	0	0
P305	Test animal	FF	Transfusion	PLT	2308	3	9	1	11
P525	Test animal	LF	Transfusion	PLS	2163	7	21	11	52
P428	Test animal	LF	Transfusion	RCC	2800	3	9	1	11
P456	Test animal	LF	Transfusion	PLS	3529	3	9	1	11

Sheep ID	Status	Codon 141 *PRNP* genotype	Route of infection	Component transfused	Survival period (days post-infection)	No. of PMCA experiments	Total no. of replicates	No. of positive replicates	% positive replicates
**Group 2**									
P208	Test animal	FF	Transfusion	BC	2304	3	9	2	22
P230	Test animal	FF	Transfusion	BC	1968	3	9	1	11
P225	Test animal	LF	Transfusion	BC	503	6	16	14	88
N480	Test animal	LF	Transfusion	BC	2177	3	9	0	0
P243	Test animal	LF	Transfusion	BC	2323	5	13	7	54
M305	Test animal	FF	Transfusion	BC	2134	3	9	4	44
P217	Test animal	LF	Transfusion	BC	3138	3	9	0	0
M538	Test animal	LL	Transfusion	BC	400	3	9	1	11
M177	Test animal	FF	Transfusion	BC	2248	5	13	3	23
M260	Test animal	FF	Transfusion	BC	981	3	9	0	0
M522	Test animal	LF	Transfusion	BC	2277	3	9	5	56
N519	Test animal	LF	Transfusion	BC	2254	3	9	0	0
P464	Test animal	LF	Transfusion	BC	2550	4	10	0	0
M518	Test animal	FF	Transfusion	BC	603	4	11	0	0
P353	Test animal	LF	Transfusion	BC	2109	5	13	13	100
M532	Test animal	LF	Transfusion	BC	2158	3	9	1	11
P522	Test animal	LF	Transfusion	PLT	1791	3	9	1	11
P279	Test animal	FF	Transfusion	PLT	1944	3	9	3	33
P275	Test animal	LF	Transfusion	PLT	587	3	9	0	0
P343	Test animal	FF	Transfusion	PLT	788	4	9	0	0
M360	Test animal	LF	Transfusion	PLT	2049	3	9	0	0
N235	Test animal	LF	Transfusion	PLT	1674	3	9	0	0
N387	Test animal	LF	Transfusion	PLT	2444	3	9	1	11
M271	Test animal	LF	Transfusion	PLT	590	3	9	3	33
P306	Test animal	LF	Transfusion	RCC	1603	3	9	0	0
N221	Test animal	LF	Transfusion	RCC	2107	3	9	0	0
P177	Test animal	LF	Transfusion	WB	2218	3	9	0	0
P248	Test animal	LF	Transfusion	WB	2102	5	13	11	85
M250	Test animal	FF	Transfusion	WB	2259	3	9	0	0

P455	Test animal	LF	Transfusion	WB	3995	4	11	1	9

**Sheep ID**	**Status**	**Codon 141 *PRNP* genotype**	**Route of infection**	**Component transfused**	**Survival period (days post-infection)**	**No. of PMCA experiments**	**Total no. of replicates**	**No. of positive replicates**	**% positive replicates**
**Group 3 (PCAPT recipients)**									
P541	Test animal	LF	Transfusion	LR-RCC	3249	4	12	0	0
Q391	Test animal	LF	Transfusion	LR-RCC	3437	6	14	6	43
Q397	Test animal	LF	Transfusion	LR-PCAPT-RCC	3476	6	17	14	82
P466	Test animal	LF	Transfusion	LR-PCAPT-RCC	2291	6	17	0	0
P467	Test animal	LF	Transfusion	LR-RCC	573	6	18	16	89
P461	Test animal	LF	Transfusion	LR-PCAPT-RCC	3456	3	9	0	0
P494	Test animal	LF	Transfusion	LR-RCC	3402	6	17	10	59
P304	Test animal	LF	Transfusion	LR-PCAPT-RCC	2200	6	18	9	50
P473	Test animal	LF	Transfusion	LR-RCC	2689	3	8	0	0
P280	Test animal	LF	Transfusion	LR-PCAPT-RCC	1736	5	16	1	6

Key:		
	WB	Whole blood
	BC	Buffy coat
	RCC	Red cell concentrate
	PLT	Platelets
	PLS	Plasma
	LR	Leucodepleted
	PCAPT	P-CAPT filtered

Experiment series 1 Groups 1 and 3
Experiment series 2 Group 2

Table S3. Results of PMCA analysis of prescapular lymph node (PSLN) and brain samples from secondary recipient sheep

Sheep ID (Primary recipient)	% positive PMCA replicates (PSLN)	Sheep ID (Secondary recipient)	*PRNP* codon 141 genotype	Survival period (dpi)	No. of PMCA experiments	PMCA results (no. of positive replicates/total number of replicates)	
						PSLN	Brain
M250	0	P308	LF	1911	2	0/5	0/5
P243	54	P500	LF	3384	2	0/5	0/5
P455	9	P538	LF	3463	2	0/5	0/5
M260	0	Q225	LF	3461	2	0/5	0/5
P177	0	Q228	LF	1858	2	0/5	0/5
P542	60	Q236	LF	3455	2	0/5	0/5
P458	NT	Q274	LF	3254	2	0/5	0/5
P248	85	Q282	LF	1718	2	0/5	0/5
P353	100	Q380	LF	1785	2	0/5	0/5
M177	23	Q387	LL	3455	2	0/5	0/5

PMCA.93 / R2 07/6/19

2 min

Uncropped blots used to create Figure 1 (A & B)

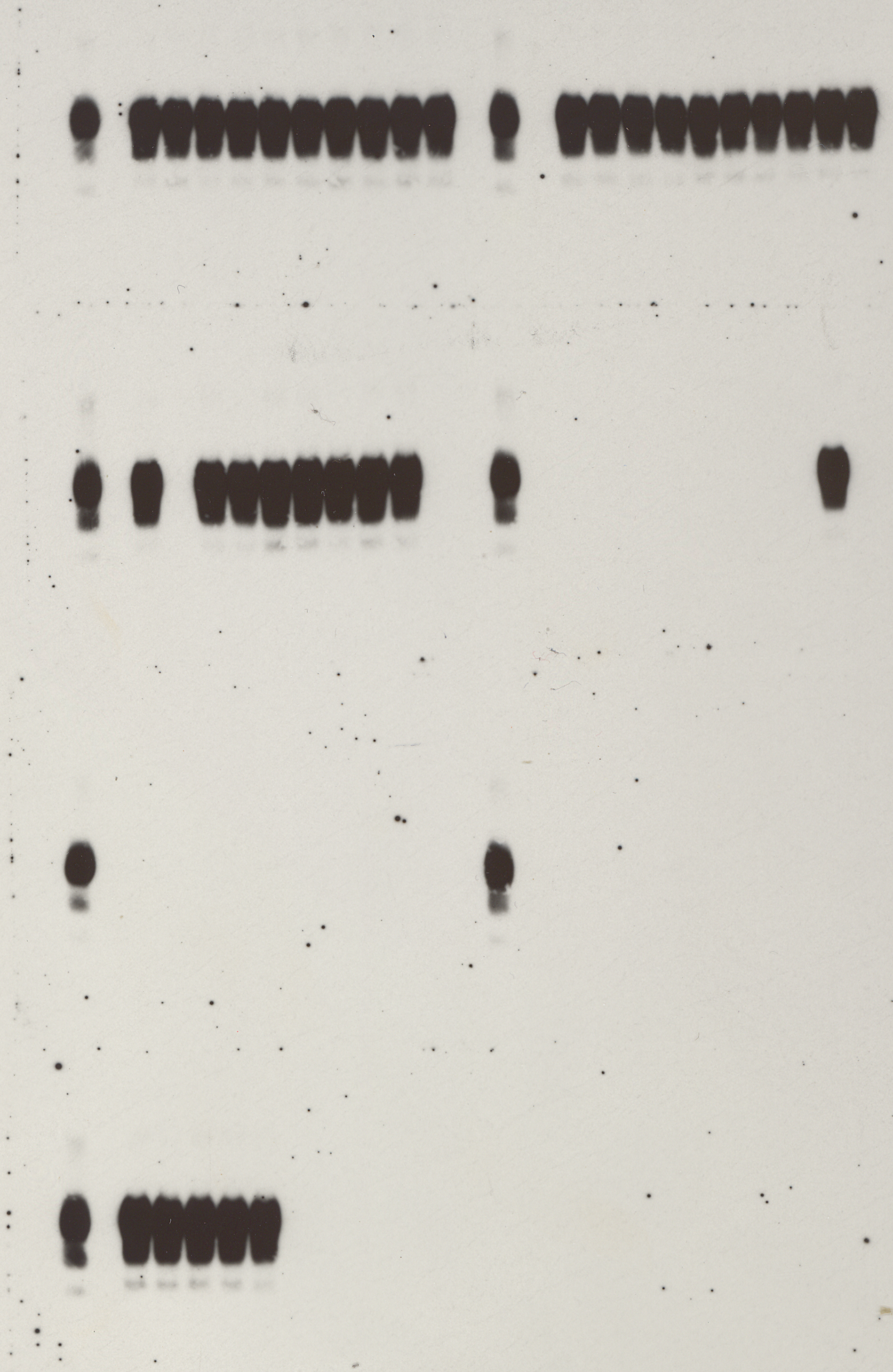

Supplement: Supplementary file 1 — Supplementary Information. [file 41598_2022_15105_MOESM1_ESM.pdf]
